# Nanoparticles Prepared From N,N-Dimethyl-N-Octyl Chitosan as the Novel Approach for Oral Delivery of Insulin: Preparation, Statistical Optimization and *In-vitro* Characterization

**Published:** 2018

**Authors:** Elnaz Sadat Shamsa, Reza Mahjub, Maryam Mansoorpour, Morteza Rafiee-Tehrani, Farid Abedin Dorkoosh

**Affiliations:** a *Department of Pharmaceutics, School of Pharmacy, Hamadan University of Medical Sciences, Hamadan, Iran. *; b *Department of Pharmaceutics, Faculty of Pharmacy, Tehran University of Medical Sciences, Tehran, Iran.*

**Keywords:** Caco-2 cell permeability, Cytotoxicity studies, N, N-Dimethyl-N-Octyl chitosan, Oral drug delivery, Insulin nanoparticles

## Abstract

In this study, N,N-Dimethyl-N-Octyl chitosan was synthesized. Nanoparticles containing insulin were prepared using PEC method and were statistically optimized using the Box-Behnken response surface methodology. The independent factors were considered to be the insulin concentration, concentration and pH of the polymer solution, while the dependent factors were characterized as the size, zeta potential, PdI and entrapment efficiency. The optimized nanoparticles were morphologically studied using SEM. The cytotoxicity of the nanoparticles on the Caco-2 cell culture was studied using the MTT cytotoxicity assay method, while the permeation of the insulin nanoparticles across the Caco-2 cell monolayer was also determined. The optimized nanoparticles posed appropriate physicochemical properties. The SEM morphological studies showed spherical to sub-spherical nanoparticles with no sign of aggregation. The *in-vitro* release study showed that 95.5 ± 1.40% of the loaded insulin was released in 400 min. The permeability studies revealed significant enhancement in the insulin permeability using nanoparticles prepared from octyl chitosan at 240 min (11.3 ± 0.78%). The obtained data revealed that insulin nanoparticles prepared from N,N-Dimethyl-N-Octyl chitosan can be considered as the good candidate for oral delivery of insulin compared to nanoparticles prepared from N,N,N-trimethyl chitosan.

## Introduction

According to the international diabetes federation, there are more than 300 million people suffering from diabetes mellitus worldwide (1); of which more than 30 million of those patients have insulin dependent diabetes mellitus (IDDM) (2). The common treatment for IDDM consists of daily subcutaneous injections of insulin, which are often difficult and inconvenient for the patient (3). Therefore, alternative non-parenteral ways, including oral delivery, pulmonary administration, transdermal delivery, intranasal, ocular, rectal, vaginal/uterine, buccal, and transmucosal routes have been considered (4). Among these different routes of administration, oral drug delivery is considered to be the most acceptable route of administration. It is non-invasive, and since insulin would be directly transferred from the intestine or colon to the liver, peripheral hyperinsulinemia could be avoided (5). Nevertheless, due to certain problems in oral administration, such as poor absorption through the gastrointestinal epithelium and rapid degradation due to enzymatic activity, the development of new strategies which can overcome these obstacles is necessary (6). Different methods have been investigated to improve the oral delivery of peptides, including the use of an absorption enhancer (7, 8), enzyme inhibitors (9, 10), lipid based vehicles (11), modifying chemical structures (12), and self-nano-emulsifying drug delivery system (13). The manipulation of absorption enhancers in the delivery system is an effective strategy to improve oral peptide drug delivery, and the suitable enhancers require mucoadhesive properties, the ability to reversibly open tight junctions, act as local enzyme inhibitors and show no toxicity (6). 

Comprehensive studies have been done using chitosan in peptide drug delivery, because it is a biodegradable cationic polysaccharide which is obtained from the deacetylation of chitin (14). Studies have shown that chitosan can help quick improvement of GI ulcers (15), has antimicrobial effects, can reversibly open tight junctions, and facilitates para-cellular absorption.

The amino group in chitosan has a pKa value of ~6.5, which leads to a protonation in acidic media with a pH dependent charge density. This makes chitosan water soluble and bioadhesive, allowing it to readily bind to negatively charged surfaces, such as mucosal membranes in acidic pH; however, it possesses low solubility in the intestinal alkaline pH. To overcome this limitation, derivatives of chitosan have been synthesized, and these quaternized derivatives pose more solubility in a wide range of pH values (16, 17). 

In 2008, Sajomsang reported the synthesis of methylated N-(4-N, N-dimethyl aminobenzyl) chitosan. In this study, an aromatic group with quaternized amine has been substituted to the primary amino group of chitosan. Gene delivery studies on this derivative show higher transfection efficiency in comparison with previously studied trimethyl chitosan (18, 19). In 2011, Mahjub *et al.* synthesized three quaternized aromatic derivatives of chitosan, including methylated N-(4-N, N-dimethylaminobenzyl) chitosan, methylated N-(4-pyridinyl) chitosan, and methylated N-(benzyl) chitosan, used for the preparation of insulin nanoparticles. They showed improvement in the oral delivery of insulin using the aromatic derivatives of chitosan due to the well-established interaction between the polymer and insulin (20). 

Over the last few decades, different delivery systems have been studied, including nanosystems, liposomes, microparticles, and ethosomes (21). Among the systems mentioned above, nanosystems have some advantages including increasing absorption due to increased gastric residence time, cell entry leading to enhanced delivery to target tissue, ability to prepare and sustain the drug release system, decrease in side effects, and reduction of clearance leading to increasing bioavailability (21). There are many techniques for preparing nanoparticles possessing solvent evaporation (22), surface polymerization (23), and emulsion polymerization (24), but most of these methods are based on the use of organic solvents, heat or extreme agitation, which may cause damage to the peptides and proteins. In recent years, the polyelectrolyte complexion method (PEC) has become preferred (25). This method does not require a harsh procedure for the preparation of nanoparticles; therefore, possible damage in the process of drug nanoparticle preparation is minimized. 

The basis of this method is via electrostatic interactions between a positively charged polymer and negatively charged insulin, developing a colloidal dispersion of nanoparticles that are stable and uniform (26). In this study, we used a long chain alkylated derivative of low molecular weight chitosan (LMWC) as a matrix material for the insulin nanoparticle preparation (14, 27). LMWC can be more effective than high molecular weight chitosan, as it is more soluble in water and can entrap insulin into its nanostructure (27). It is assumed that a long chain alkylated chitosan (*i.e.* methylated (N-octyl) chitosan) has the ability to improve epithelial absorption due to its higher hydrophobicity when compared with trimethyl chitosan (20). 

## Experimental

*Materials*

Low molecular weight chitosan was supplied from Primex (Iceland). Sodium iodide, iodomethane, triethylamine, N-Methyl pyrolidone (NMP) and sodium hydroxide were provided from Merck (Darmstadt, Germany). Sodium borohydride and octanal were provided from Sigma (UK). Human insulin was obtained from Ronak Pharmaceutical Co. (Saveh, Iran). Dialysing tube with a molecular cut off of 12,000 Da (D0405) was obtained from Sigma (UK). Caco-2 cell line with passage number 30-40 was provided from Pasteur institute (Tehran, Iran). Analytical grade acetonitrile were obtained from Merck (Darmstadt, Germany). All other chemicals were of pharmaceutical grade and used as received.

*Synthesis and characterization of N,N-Dimethyl-N-Octyl chitosan*

In this study, a Schiff based reaction was used to add a long chain hydrocarbon (octyl) to chitosan (28). For the synthesis of the proper derivative, 5 g of chitosan was dissolved in 1% acetic acid (v/v). After complete dissolution of the octanal, 12.5 mL were added and diluted with methanol (100 mL) to reduce the viscosity. The pH was adjusted to 5.0 using sodium hydroxide, and kept under stirring for 24 h. The appropriate imine was reduced using sodium borohydride (8 g) and then the solution was kept under stirring for an additional 12 h. The N-octyl derivative of chitosan was precipitated by increasing the pH to 9.0. The precipitate was then washed with methanol, dissolved in 1% acetic acid and dialyzed for 3 days against distilled water using a dialyzing tube. The desired derivatives were then precipitated using NaOH (1 N), and the precipitates were transferred to a vacuum dryer and placed overnight for complete drying. 

The precipitate prepared in the previous step (2 g) was dissolved in NMP; then, methyl iodide (10 mL) and sodium iodide (2 g) were added to perform the N-methylation reaction (29). The solution was refluxed at 50 °C overnight, after which the solution was transferred to a dialyzing tube and dialyzed against distilled water for 3 days. The appropriate compound was precipitated by adding 100 mL of acetone. In order to exchange the I^-^ with Cl^-^, the precipitated derivative was dissolved in a NaCl solution (5%, w/v) and stirred for 1 h at ambient room temperature. Then, the chloride salt of the desired compounds was precipitated. The precipitates were transferred to a vacuum dryer and held overnight for complete drying. The quaternized derivatives were characterized by ^1^H-NMR. Finally, the degree of substitution and degree of quaternization were also determined from integrated data obtained from the ^1^H-NMR spectra.

**Figure 1:**
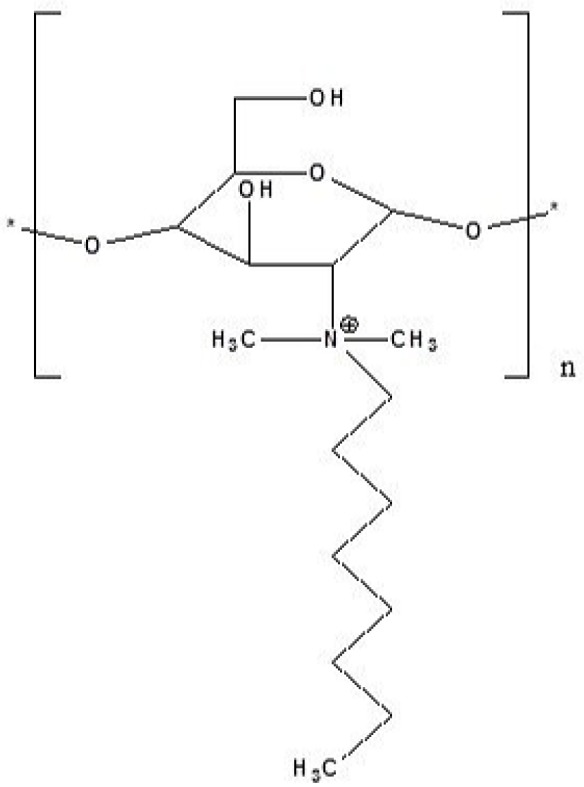
Chemical Structure of N,N-Dimethyl-N-Octyl chitosan

The molecular structure of the synthesized derivative is shown in Figure 1.

*Preparation of nanoparticles*

Nanoparticles were prepared by poly electrolyte complexation known as PEC (30, 31). In this method particle preparation is based on electrostatic interaction between opposite charges. Briefly, polymer solution with positive charge, and insulin solution with negative charge were separately prepared and filtered through 0.4 µm pore size filter. Finally, insulin nanoparticles were prepared by gradual adding insulin solution (2 mL) to the equal volume of polymer solution at ambient temperature under certain stirring speed (500 rpm) at room temperature. The formation of opalsant colloidal suspension indicated the preparation of nanoparticles. For better electrostatic interaction, the colloidal nano-suspension was kept stirred for 20 min. 

After formation of nanoparticles, the colloidal suspension was centrifuged at 14,000 rpm in 4 °C for 20 min. The supernatant was separated and collected for further analysis. Settled down nanoparticles were re-suspended in deionized water. In this study, Box-Behenken response surface methodology was used for statistical optimization of nanoparticles. 

*Characterization of nanoparticles*

*Determination of size and zeta potential of the particles*

Size of nanoparticles were determined by photon correlation spectroscopy (PCS) technique using Zetasizer 300HS (Malvern instruments, Malvern, UK). Nanoparticles were diluted at 1:5 ratio by pre-filtered de-ionized water (32). Zeta potential of the particles was determined using laser doppler anemometry by the same instrument, mentioned above. All measurements were done in triplicate. 

*Determination of entrapment efficiency (EE%) and loading efficiency (LE)*

For determination of EE% and LE%, the freshly prepared colloidal suspensions were centrifuged at 14,000 rpm for 20 min and the supernatant was analyzed for determination of non-encapsulated insulin using HPLC. 

The samples were injected to Agilent® 1260 infinity equipped with 1260 Quat pump VL, 1260 ALS auto sampler, and 1260 DAD VL detector that was set at 214 nm. MZ^®^analytical Perfect Sil Target^®^ODS−3 (150 × 4.6 mm, 5 µm) C18 column was used for HPLC analysis of insulin. Mobile phase was obtained from a mixture of buffer: acetonitrile in ratio of 70:30. Buffer was prepared from KH_2_PO_4 _(0.1 M) and triethylamine (1%) and the pH was adjusted to 2.8 using phosphoric acid. 

The flow rate was set on 0.5 mL/min and the data were captured using Agilent ChemSation® software. The HPLC method was partially validated according to ICH guidelines and also linearity, intra- day and inter- day precision and accuracy, limit of detection (LOD), and limit of quantification (LOQ) were determined. The calibration curve was showed to be linear (R^2 ^= 0.998) over concentration range of 0.5 µg/mL to 20 µg/mL. The values of RSD% and Error%, as the indexes for determination of precision and accuracy are respectively shown on Tables 1 and 2. LOD and LOQ were determined by signal to noise ratio and reported as 0.1 µg/mL and 0.3 µg/mL, respectively.

EE% and LE% were calculated by determination of non-encapsulated insulin in the supernatant of previously centrifuged nano-suspension. Equation 1 and Equation 2 were used to calculate the entrapment efficiency and loading efficiency, respectively.

$\mathrm{EE\%}=\frac{\mathrm{totalamountofinsulin}-\mathrm{amountofinsulininsupernatant}}{\mathrm{totalamountofinsulin}}\times 100$

 (Equation 1)

$\mathrm{LE\%}=\frac{\mathrm{totalamountofinsulin}-\mathrm{amountofinsulininsupernatant}}{\mathrm{totalweightofnanoparticle}}\times 100$

(Equation 2)

*Statistical optimization of physicochemical properties of nanoparticles*

In this study, the physicochemical properties of the nanoparticles were optimized using the Box-Behnken response surface methodology by Design-Expert® software (V. 7.0.0, Stat-Ease, Inc., Minneapolis, USA). Independent variables (factors), including insulin concentration (X_1_), polymer concentration (X_2_), and the pH of the polymer solution (X_3_) were determined using previous screening tests. 

The dependent variables (Responses) were considered as the size (Y_1_), zeta potential (Y_2_), polydispersity index (PdI) (Y_3_), and entrapment efficiency (EE%) (Y_4_). The ranges of the defined factors are illustrated in Table 3. Based on the statistical calculation, 17 experiments were required for the development of an appropriate model (Shown in Table 4). In all studies, the pH of the insulin solution was adjusted to 8.0 as the constant factor. The obtained experimental data were analyzed using Design-Expert®, and the developed model was described using the mathematical equation. Finally, the predicted models were explained by second-order functions as 

follows:

Y = β_0 _+ β_1_ A + β_2_ B + β_3_ C + β_11_ A^2^ + β_22_ B^2 ^+ β_33 _C^2 ^+ β_12_ A.B + β_13_ A.C + β_23_ B.C

Where: 

Y: predicted response for size, zeta potential, PdI and EE%;

β_0_: Intercept;

β_1_, β_2_ and β_3_: Linear coefficients;

β_11_, β_22_ and β_33_: Square coefficients;

β_12_, β_13_ and β_23_: Interaction coefficient;

A, B and C: Independent variables;

*Freeze drying of optimized nanoparticles*

The optimized nanoparticles were centrifuged and settled down nanoparticles were re-suspended in distilled water, then freeze dried. For the lyophilization process, the nanoparticles were freezed at -20 ºC for 24 h. Freeze drying was performed using Christ^®^ (Martin Christ GmbH, Osterode am Harz, Germany). For freeze drying, samples were dried for 48 h at a working pressure of 0.07 mbar at the condenser temperature of -50 ºC. The sucrose (5% w/v) was used as the lyoprotectant for lyophilization process. For evaluation of the stability of nanoparticles during lyophilization, freeze- dried nanoparticles were suspended in doubled distilled water that was previously filtered through 0.22 µm syringe filter and the physico-chemical properties of the colloidal suspension were determined.

*Morphological study of nanoparticles*

For determination of morphology, a fresh sample of lyophilized optimized nanoparticle suspension was prepared and diluted at 1:5 ratio using freshly prepared double distilled water. One drop of colloidal nano-suspension was placed on a slide and was left to dry. The samples were coated with gold and were examined by scanning electron microscopy (SEM).

*In-vitro release studies*

The release of insulin from lyophilized nanoparticles was studied in simulated intestinal fluid. The medium was prepared by dissolving KH_2_PO_4_ (6.8 g) in 250 mL de-ionized water, then 77 mL of sodium hydroxide (0.2 M) was added and the volume was brought to 1000 mL. The pH was adjusted to 6.8.

For* in-vitro* release studies, proper amount of lyophilized nanoparticles equivalent to 50 mg of insulin was suspended in 100 mL of SIF, shaking at 50 rpm. The temperature was set constant at 37 ± 1 °C. The conditions were chosen so as to ensure establishment of sink condition. At predetermined post incubation time intervals (*i.e.* 0, 15, 30, 45, 60, 90, 120, 150, 180, 225, 270, 330 and 390 min), samples (1 mL) were collected and replaced by pre-heated blank medium. The samples were centrifuged at 12,000 rpm for 20 min. The amount of insulin in the supernatant was determined using previously mentioned HPLC.

*Caco-2 cell culture*

Caco2 cells were provided from Pasteur Institute (Tehran, Iran) at passage number of 30-40. Cells were cultured on 25 cm^2 ^Nunc plastic flasks (Roskilde, Denmark). The medium was contained modified eagle medium (MEM) supplemented with 1% v/v non-essential amino acids (90 v/v), fetal bovine serum (FBS, 9% v/v), and penicillin-streptomycin (100 U/mL, 1% v/v). The cells were incubated in a humidified atmosphere containing 5% CO_2_ and 95% air at 37 °C. The culture medium was changed every second day. The cells were passaged after 7 days when reaching to desired confluency. 

*Evaluation of cytotoxicity*

The cytotoxic effects of prepared nanoparticles were studied on Caco-2 cells using MTT cell cytotoxicity assay. The cells were seeded on a 96-well cell culture plate provided from Nunc (Roskilde, Denmark) at a density of 1 × 10^4^ cells per well and pre-incubated for 24 h to ascertain cell attachment. Then, the cells were exposed to the prepared nanoparticles at various concentration equivalents of 0.01 to 5 mM of polymer in white modified eagle medium containing sterilized water (500 mL), glutamine (5 mL), and sodium bicarbonate (1.1 g) buffered with n-(2-hydroxyethyl) piperazine-n-(2-ethanosulfonic acid) (HEPES) at pH 7.4 for 5 or 24 h. The concentration range of polymer was chosen based on preliminary studies in a manner that cell viability above 90% as well as below 50% could be observed and therefore the related data would be appropriate for calculation of IC_50_. After treatment, the medium was suctioned and the cells were washed with phosphate buffer saline (PBS). Thirty micro liter of MTT (5 mg/mL) were added to each well, and cells were incubated for 4 h until formazan crystals in living cells appeared. The crystals were dissolved in dimethyl sulfoxide (DMSO) and the absorbance was determined at 550 nm using an ELISA microplate reader. 

The non-treated cells are considered to be the control and their related viability was assumed to be 100%. The related IC_50_ was calculated by the curve fitting of the cell viability data using Prism^®^ software (V. 4.0 GraphPad, San Diego, CA). 

**Table 1:** Intra-day precision and accuracy (n = 3).

Concentration added (µg/mL)	Concentration found (µg/mL)Mean ± SD	RSD (%)	Error (%)
0.5	0.52 ± 0.02	3.84	4.00
1	1.06 ± 0.06	5.60	6.00
5	4.96 ± 0.06	1.20	-0.8
10	10.15 ± 0.14	1.37	1.50
20	20.67 ± 0.53	5.03	3.25

**Table 2:** Inter-day precision and accuracy (n = 3).

**Concentration added ** **(µg/mL)**	**Concentration found (µg/mL)** **Mean ± SD**	**RSD (%)**	**Error (%)**
0.5	0.46 ± 0.05	10.86	-8.00
1	1.11 ± 0.09	8.10	11.00
5	5.26 ± 0.14	2.66	5.20
10	10.38 ± 0.36	3.50	4.73
20	21.74 ± 2.35	10.7	8.80

**Table 3:** Variables used in Box-Behenken Response Surface Methodology

**Independent variables (Factors)**			**Levels**	
			**-1**	**+1**
Numeric factors	Polymer pH (A)		3.0	6.0
	Polymer Concentration (B)		0.5	2.0
	Insulin Concentration (C)		0.5	2.0
**Dependent variables (Responses)**		**Constrains**		
Y_1 _= Size (nm)		Minimize		
Y_2 _= PdI		Minimize		
Y_3 _= Zeta Potential		Maximize		
Y_4 _= Entrapment Efficiency (EE%)		Maximize		

**Table 4:** Box-Behenken experimental design runs (n = 3).

**Formulation number**	**Independent Variables**			**Dependent Variables**			
	**Polymer pH (A)**	**Polymer concentration (B)**	**Insulin concentration (C)**	**Size (Y**_1_ **) (nm)** **Mean ± SD**	**Zeta (Y**_2_ **) (mV)** **Mean ± SD**	**PdI (Y**_3_ **)** **Mean ± SD**	**EE (Y**_4_ **) (%)** **Mean ± SD**
1	4.50	1.25	1.25	902 ± 28.3	17.8 ± 2.64	0.807 ± 0.06	76.4 ± 4.11
2	4.50	1.25	1.25	519 ± 16.7	13.1 ± 0.61	0.642 ± 0.08	63.7 ± 3.95
3	4.50	1.25	1.25	230 ± 32.3	15.8 ± 3.19	0.900 ± 0.08	71.1 ± 2.74
4	4.50	1.25	1.25	1148 ± 35.9	16.3 ± 1.53	0.878 ± 0.05	70.5 ± 1.61
5	3.00	1.25	0.50	150 ± 18.5	17.5 ± 1.40	0.451 ± 0.03	56.6 ± 2.24
6	3.00	1.25	2.00	959 ± 36.9	15.4 ± 2.75	0.753 ± 0.08	90.8 ± 3.15
7	6.00	1.25	2.00	225 ± 18.5	12.3 ± 2.64	0.830 ± 0.05	89.9 ± 4.32
8	4.50	0.50	2.00	986 ± 46.8	15.1 ± 0.90	0.910 ± 0.09	97.1 ± 1.94
9	6.00	0.50	1.25	985 ± 27.4	10.6 ± 1.27	0.9 ± 0.07	42.9 ± 2.28
10	3.00	2.00	1.25	1846 ± 53.8	17.2 ± 0.55	0.9 ± 0.08	56.6 ± 3.72
11	4.50	1.25	1.25	991 ± 37.5	13.9 ± 1.82	0.771 ± 0.05	72.8 ± 3.26
12	3.00	0.50	1.25	388 ± 35.2	17.2 ± 2.67	0.525 ± 0.04	54.1 ± 1.90
13	6.00	1.25	0.50	545 ± 67.2	11.5 ± 1.06	0.575 ± 0.04	26.1 ± 2.15
14	4.50	2.00	2.00	1603 ± 46.2	16.7 ± 0.74	0.950 ± 0.07	87.2 ± 3.54
15	6.00	2.00	1.25	260 ± 11.7	16.4 ± 1.17	0.512 ± 0.03	81.3 ± 4.37
16	4.50	0.50	0.50	360 ± 25.3	16.4 ± 2.07	0.696 ± 0.06	17.0 ± 2.83
17	4.50	2.00	0.50	1577 ± 48.2	25.3 ± 2.18	0.988 ± 0.08	66.3 ± 3.74

**Table 5:** Characteristics of synthesized N,N-dimethyl, N-octyl chitosan

**Polymer Type**	**Degree of N-alkyl Substituation (DS) %**	**Degree of Quaternization (DQ) %**	**Recovery (%)**
N,N-Dimethyl-N-Octyl chitosan	26.4%	43.6%	78.9

**Table 6:** Characteristics of the models fitted to responses

**Dependent variables (Responses)**	**Best fitted model**	**R-squared**	**Adj R-squared**	**Pred R-square**	**Adeq precision**
Size (Y_1_)	Quadratic	0.6288	0.5051	0.2179	8.583
Zeta potantial (Y_2_)	Quadratic	0.7952	0.6431	0.5219	5.58
PdI (Y_3_)	Quadratic	0.735	0.6145	0.3371	8.724
EE% (Y_4_)	Quadratic	0.9669	0.9470	0.8821	24.055

**Table 7:** Optimized independent variables and the related predicted values.

**No.**	**Optimized independent variables**			**Predicted dependent variables (Responses)**			
	**Polymer pH (A)**	**Polymer concentration (B)**	**Insul** **in concentration (C)**	**Y**_1 _ **= Size** **(nm)**	**Y**_2 _ **= Zeta potential (mV)**	**Y**_3 _ **= PdI**	**Y**_4 _ **= EE (%)**
1	3.0	0.5	1.98	304	16.9	0.324	97.1

**Table 8:** Observed Responses and Prediction Errors (n = 5).

**Size (nm)**		**Zeta Potential ( mV)**		**PdI**		**EE%**		**LE (%)**
Observed response (Mean ± SD)	Prediction Error (%)	Observed response (Mean ± SD)	Prediction Error (%)	Observed response (Mean ± SD)	Prediction Error (%)	Observed response (Mean ± SD)	Prediction Error (%)	Observed responses (Mean ± SD)
334 ± 25.3	9.78-%	17.2 ± 3.1	1.59	0.317 ± 0.04	-2.16%	90.8 ± 2.51	-6.48	9.3 ± 1.7

**Table 9:** Physico-chemical properties of nanoparticles after lyophilization (n = 5).

**Size (nm)** **(Mean ± SD)**	**Zeta potential (mV)** **(Mean ± SD)**	**PdI ** **(Mean ± SD)**	**EE (%)** **(Mean ± SD)**	**LE%** **(Mean ± SD)**
367 ± 42.6	11.6 ± 2.4	0.321 ± 0.03	83.1 ± 3.42	6.7 ± 2.41

**Figure 2:**
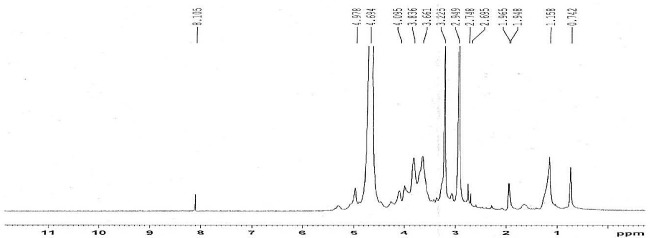
H-NMR Spectrum of N,N-Dimethyl-N-Octyl chitosan

**Figure 3:**
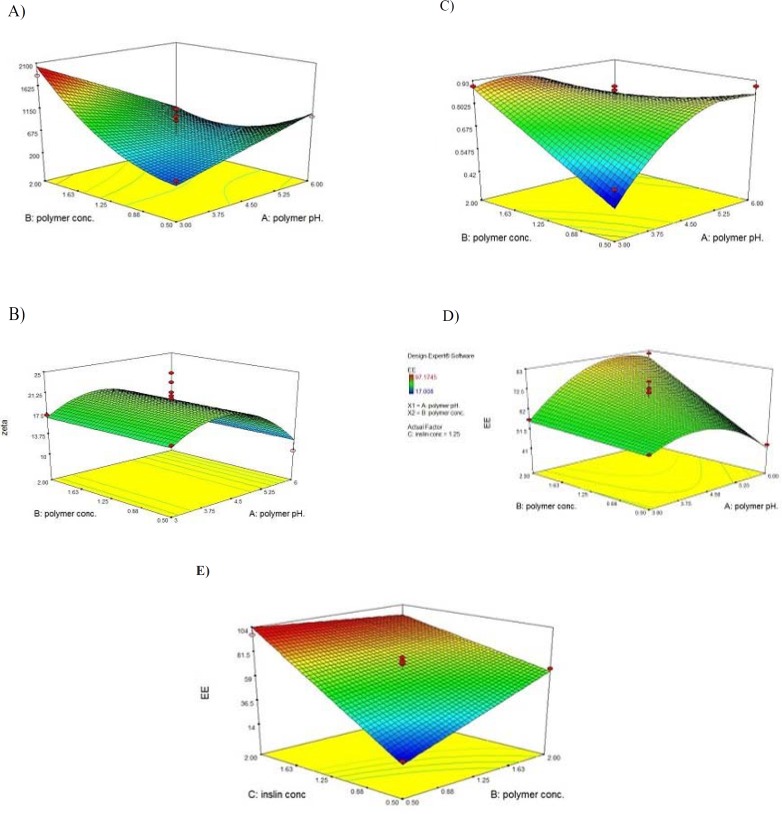
3D Reponse surface plots for (A) Size; (B) Zeta Potential; (C) PdI; (D, E) EE%.

**Figure 4:**
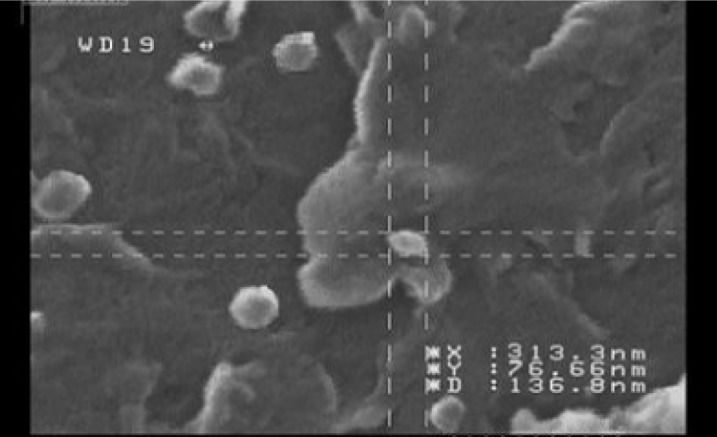
Morphological images acquired by SEM.

**Figure 5:**
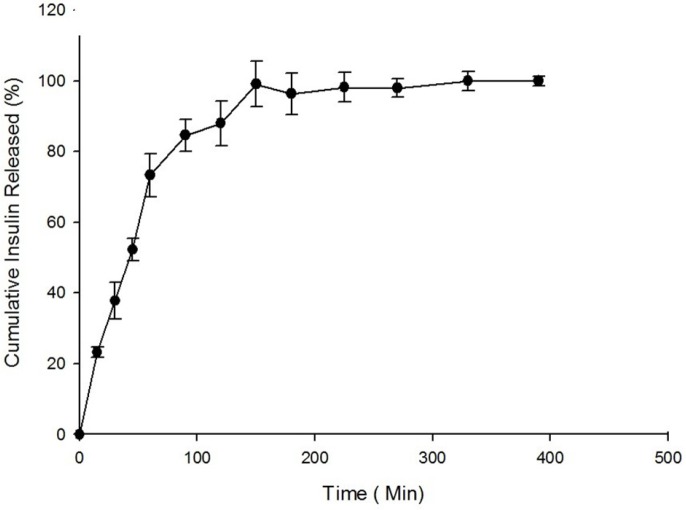
*In-vitro *Release profile of Insulin from Nanoparticles (n = 3).

**Figure 6:**
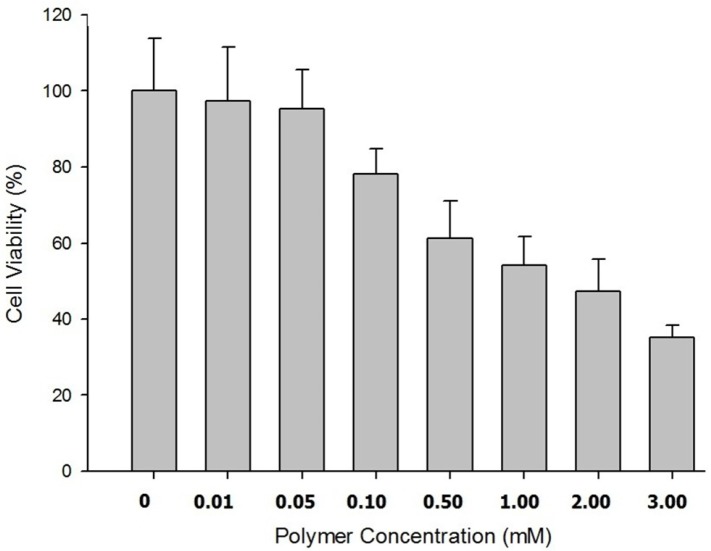
Cell viability of Caco-2 cell cultrure 24 h post incubation.

**Figure 7:**
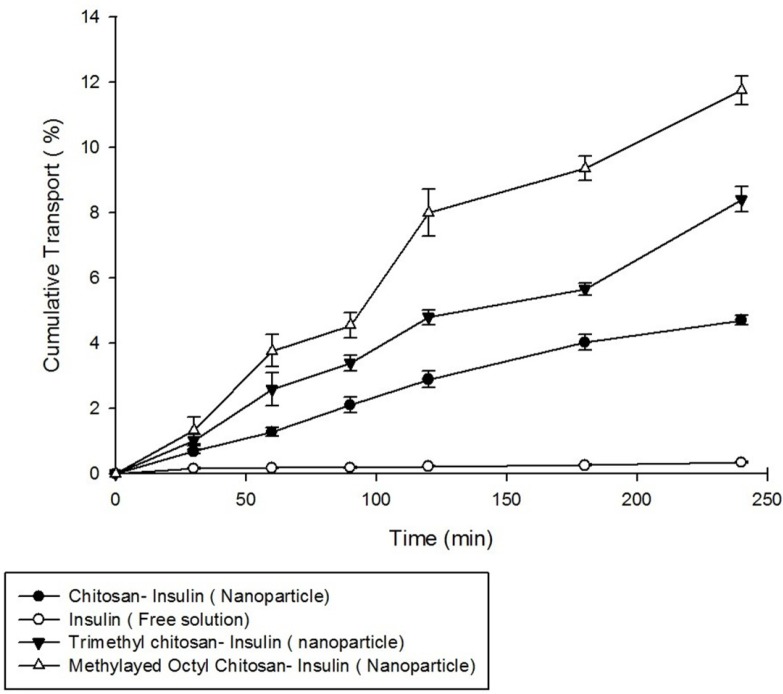
Cumulative Insulin trasnported across Caco-2 cell monolayer (n = 3).

*Permeability studies*

For performing the permeability studies on Caco-2 cell monolayer, insulin was used in free solution form and in the form of nanoparticulatecolloidal suspensions prepared from non-modified chitosan, trimethyl chitosan (TMC), and methylated N-octyl chitosan. 

Caco-2 cells with passage number of 30-45 were obtained as mentioned before. The cells were seeded on polyethylene terephthalate (PET) membrane filters with pore size 0.4 µm (Grenier Bio one, Manore, NC, US) in 12-well plate at the cell density of 2 × 10^4^ cell/cm^2^. The medium included modified eagle medium mentioned previously in this publication. The culture medium was added to both apical (1 mL) and basolateral (2 mL) compartments and was changed every second day for 18 days. The cells were incubated at 37 ºC in an atmosphere of 5% CO_2 _at 90% humidity. To ensure the formation of cell monolayer, the transepithelial electrical resistance (TEER) was determined every second day using an EVOM^2^, epithelial voltammeter for TEER (World Precision Instruments, Sarasota, FL, US) equipped with chopstick electrode set. The experiment was performed when the TEER values were more than the values of 600-700 Ω cm^2^ for ensuring formation of a monolayer. On the other hand, the transwell membrane was studied visually by optical microscopy for ensuring the formation of a monolayer.

One hour before the experiment, the medium in both apical and basolateral sides was changed to a transport medium: transparent MEM buffered with n-(2-hydroxyethyl) piperazine-n-(2-ethanosulfonic acid) (HEPES) at pH 7.4, and the cells were allowed to equilibrate for 1 h. 

For permeability studies, 1 mL of 0.40 mg/mL final concentration of insulin in the form of nanoparticle suspensions prepared were added to the apical side of the cell culture dish. The samples of 50 µL were collected from the basolateral part at predetermined times of 0, 30, 60, 90, 120, 180, and 240 min and replaced with equal volumes of fresh transparent MEM-HEPES medium. The samples were analyzed for the insulin content using previously mentioned HPLC method. The study was performed in triplicate.

*Statistical analysis*

All experiments were performed in triplicate and the related values were reported as mean ± SD. Statistical significance of differences was evaluated by one way analysis of variance (ANOVA) with appropriate post hoc tests using SPSS^®^ (V. 19.0.0, IBM Statistics, New York, USA). The differences were considered significant when *p *< 0.05. 

Design-Expert® software ( V. 8.0.7.1, Stat-Ease, Inc., Minneapolis, USA) associated with appropriate ANOVA studies has been used for mathematical modeling in response surface methodology. 

## Results

*Synthesis and characterization of N,N-Dimethyl-N-Octyl chitosan*

The ^1^H-NMR spectrum of methylated N-Octyl chitosan is shown in Figure 2; where the peaks at 2.9 and 3.2 ppm represent the protons of the methyl groups of the [-N(CH_3_)-] and [-N^+^(CH_3_)_2_-] in the methylated octyl chitosan, indicating the formation of a quaternized derivative. The peaks at 3.2 to 4.8 indicate hydrogens of the sugar skeleton of chitosan, and the peaks at 4.9 to 5.1 are related to the anomeric protons of the sugar. The peaks falling below 1 ppm show the hydrogens of the terminal methyl group of octyl, and peaks at 1.5 to 1.9 are related to the hydrogens of the CH_2_ of the octyl chain, indicating the formation of the octyl derivative of chitosan. The characteristics of the synthesized derivative have been summarized in Table 5.

*Statistical optimization of physicochemical properties of nanoparticles*

For the statistical analysis, the Box-Behnken response surface methodology was used to determine the effects of the independent variables, including the insulin concentration, polymer concentration, and pH of the polymer solution, as well as the size, zeta potential, PdI, and EE% of the prepared nanoparticles. This methodology can predict the response by fitting the obtained data to the appropriate mathematical models, and finally, optimize the physicochemical properties of the nanoparticles. The designed Box-Behnken runs are summarized in Table 4. All experiments were done in triplicate.

*Size of nanoparticles*

As shown in Table 4, the size of the nanoparticles ranged between 150 ± 18.5 nm to 1846 ± 53.7 nm in the different Box-Behnken runs. Suggesting formulations are shown in Table 4. 

Based on the ANOVA statistical analysis performed using the Design-Expert software, it was found that the identified model is significant (*p < 0.01*) and the two of main factors (*i.e.*, concentration of polymer and pH of polymer solution) have significant influences on the size of the nanoparticles (*p < 0.05*). The 3-D response surface graph of the size variation as a result of the changes in the independent variables in the studied formulations is shown in Figure 3A. Significant interactions between the effects of the pH of the polymer solution, and the concentration of the polymer on the size of the particles have been determined. 

At a lower pH of the polymer solution, the size of particles would be sharply increased by increasing the polymer concentration, while in the high pH values, some decrease in particle size followed by increasing polymer concentration has been observed. 

At the lower values for the concentration of the polymer, the size of the particles was gradually increased by increasing the pH of the polymer solution. However, at the high values for the concentration of the polymer, the size of the particles was sharply decreased as a result of the increasing pH of the polymer solution. 

A statistical analysis using the Design-Expert software, based on the Box-Behnken response surface methodology, was performed, and the best significant fitted model for the particle size prediction (*p *< 0.01) was established. The characterization of the fitted model has been provided in Table 6. The regression analysis of variance for the data showed that the linear coefficients of the two main independent factors (*i.e.* A and B), the squared coefficient of B, and the interaction coefficient of A.B were significant (*p *< 0.05). The coefficient of the significant variables is shown in Equation 3, as follows:

(Equation 3) Size = -1104.36547 + 495.50932 × A + 962.09785 × B - 484.95174 × A.B + 659.16822 × B

Where:

A: pH of polymer solution;

B: Concentration of polymer;

A.B: Interaction between pH of polymer solution and concentration of polymer

B^2^: the square root of concentration of polymer;

*Zeta potential analysis*

Zeta potential indicates system stability. Particles repulsive forces are increased and particles accumulation tendency is decreased by increase in zeta potential (33). As stated in Table 4, the zeta potential of the nanoparticles varied in the range of +10.6 ± 1.27 mV to +25.3 ± 2.18 mV. 

Based on the ANOVA statistical analysis performed using the Design-Expert software, it was found that the identified model was significant (*p < 0.01*) and the only one of the main factors (*i.e.*, pH of polymer solution) that had some significant influence on the zeta potential of the nanoparticles (*p < 0.05*). The 3-D response surface graph of the variation in the zeta potential of the particles as a result of the changes in the independent variable in the studied formulations is shown in Figure 3B. No Significant interactions between the main factors were found to be significant (*p > 0.05*).

As shown in Figure 3, by increasing the pH of the polymer solution, the zeta potential of the nanoparticles was increased until reaching a specified point. By further increasing the pH of the polymer solution, the zeta potential was observed to sharply decrease due to a decrease in the positive charge density of the polymer. 

A statistical analysis using the Design-Expert software, based on the Box-Behnken response surface methodology, was performed, and the best significant fitted model for the prediction of the zeta potential (*p *< 0.01) was established. The characterization of the fitted model has been provided in Table 6. The regression analysis of the variance for the data showed that only one independent factor (*i.e*., A), the squared coefficient of A, was significant (*p *< 0.05). The interaction coefficient between the main factors was found to be non-significant (*p* > 0.05), and the coefficient of the significant variables is shown in Equation 4, as follows:

(Equation 4) Zeta Potential = -20.18467 + 19.20158 × A -2.28554 × A^2^

Where:

A: pH of polymer solution;

A^2^: the square root of pH of polymer solution;

*Polydispersity index analysis*

As shown in Table 4, the PdI varies from 0.4515 ± 0.03 to 0.988 ± 0.08. It should be noted that the PdI is an index that expresses nanoparticles homogeneity, and in terms of a numerical value, varies between zero and one.

Based on the ANOVA statistical analysis performed by the Design-Expert software, it was found that the identified model is significant (*p < *0.01) and all the main factors (*i.e.*, concentration of polymer, pH of polymer solution, and concentration of insulin) have some significant influence on the PdI of the nanoparticles (*p < *0.05). The 3-D response surface graph of the PdI variation as a result of the changes in the independent variables in the studied formulations is shown in Figure 3C. Significant interactions between the effects of the pH of the polymer solution and the concentration of the polymer on the PdI of particles have been determined. 

At lower values for the concentration of the polymer, the PdI of the particles was observed to be increased by increasing the pH of the polymer solution. Otherwise, at high values for the concentration of the polymer, the size of the nanoparticles was observed to be gradually decreased, followed by increasing the pH of the polymer solution. 

A statistical analysis using the Design-Expert software and based on the Box-Behnken response surface methodology was performed, and the best significant fitted model for the PdI prediction was established (*p *< 0.01). The characterization of the fitted model has been provided in Table 6, and a regression analysis of variance for the data has shown that the linear coefficients of the three main independent factors (*i.e.*, A, B, and C), the squared coefficient of A, and the interaction coefficient of A.B were significant (*p *< 0.05).The coefficient of the significant variables is shown in Equation 5, as follows:

(Equation 5) PdI = -1.81660 + 0.85541 × A + 0.81467 × B + 0.12203 × C.-0.16926 × A.B - 0.069795 × A^2^

Where:

A: pH of polymer solution;

B: Concentration of polymer;

C: Concentration of insulin;

A.B: Interaction between pH of polymer solution and concentration of polymer; 

A^2^: The square root of pH of polymer solution

*Entrapment Efficiency (EE%)*

As described in Table 4, the EE% varies between 17.0 ± 2.83% to 97.1 ± 1.94% in different formulations suggested by the Box-Behnken design. 

Based on the ANOVA statistical analysis performed using the Design-Expert software, it was found that the identified model is significant (*p < 0.01*) and all of the main factors (*i.e.*, concentration of polymer, pH of polymer solution, and concentration of insulin) have some significant influence on the size of the nanoparticles (*p < 0.05*). The 3-D response surface graph of the variation in EE% as a result of the changes in the independent variables in the studied formulations is shown in Figure 3D. As found, two significant interactions between the effects of the pH of polymer solution and the concentration of the polymer, as well as the interaction between the effects of the concentration of the polymer and the concentration of the insulin on the size of the particles have been determined. 

At lower values for the polymer concentration, by increasing the pH of the polymer solution, the EE% was observed to increase until a favorable value was obtained. By further increasing the pH of the polymer solution, the EE% was observed to sharply decrease due to the decreasing positive charge density in the polymer, and consequently electrostatic interaction between the polymer and the insulin. At higher values for the polymer concentration, the EE% was observed to gradually increase by increasing the pH of the polymer solution.

It was observed that the EE% was increased followed by an increase in the concentration of insulin. At lower values for the concentration of insulin, the EE% was observed to increase by increasing the concentration of the polymer, while at higher values for the concentration of insulin, a slight decrease in the EE% was observed followed by an increase in the concentration of the polymer (Figure 3E). 

A statistical analysis using the Design-Expert software and based on the Box-Behnken response surface methodology was performed, and the best significant fitted model for the prediction of EE% (*p* < 0.01) was established. The characterization of the fitted model has been provided in Table 6. The regression analysis of variance for the data has shown that the linear coefficients of all three main factors (*i.e.*, A, B, and C), the squared coefficient of A, and the interaction coefficients of A.B and B.C were significant (*p <* 0.05). The coefficient of significant variables is shown in Equation 6, as follows:

(Equation 6) EE= -108.18916 + 39.11776 × A + 10.46683 × B + 72.74772 × C + 7.97330 × -26.35635 × B.C - 5.25153 × A^2^

Where:

A: pH of polymer solution;

B: Polymer concentration;

C: Insulin concentration;

A.B: Interaction between pH of polymer solution and polymer concentration; 

B.C: Interaction between polymer concentration and insulin concentration; 

A^2^: The square root of pH of polymer concentration;

*Optimization of nanoparticles and model validation*

The physico-chemical properties of nanoparticles were statistically optimized based on response surface methodology. Box-Behnken design was used for model prediction and optimization.

Table 7 summarizes the optimized conditions for prepration of nanoparticles while the observed responses followed by predicted error values have been indicated in Table 8. As shown in Table 6, the calculated prediction errors were below 8.00% for all conditions. This represents the adequacy and predictability of models.

The physic-chemical properties of nanoparticles after lyophilization were shown on Table 9. Although after lyophilization the size and PdI of nanoparticles were slightly increased but statistical analysis showed no significant difference in size and PdI nanoparticles before and after lyophilization (*p* > 0.05). Zeta potential, EE%, and LE% of particles after lyophilization showed significant decrease compared to appropriate values before lyophilization (*p <* 0.05).

*Morphology of nanoparticles*

Investigation of the morphology of the nanoparticles by SEM has shown images with spherical to sub-spherical nanoparticles with smooth surfaces (Figure 4). As shown in the Figure 4, no sign of aggregation has been observed after lyophilization that indicates good stability of prepared nanoparticles. The data obtained from SEM images were well in accordance with the data obtained from particle size analysis using PCS technique (Malvern Instruments, Malvern, UK).

*In-vitro release study*

The *in-vitro* release of insulin from freeze-dried nanoparticles was studied in simulated intestinal fluid (SIF) while the pH was adjusted to 6.8 according to USP. The *in-vitro* drug release profile is summarized in Figure 5. As shown in Figure 5, the value of 37.74 ± 5.16% of the total loaded insulin was observed to be released at 30 min. 

According to previous studies, the cumulative amount of drug released from nanoparticles at 30 min after incubation is considered to be the burst release. Our previous studies revealed a higher release rate and burst effect for nanoparticles after lyophilization compared to nanoparticles before lyophilization due to surface migration of the drug in the nanoparticles. As illustrated in Figure 5, the total cumulative percent of insulin released from nanoparticles after 390 min of incubation is reported to be 95.5 ± 1.40%.

*Cytotoxicity assay using MTT*

The cytotoxicity of synthesized prepared nanoparticles on Caco-2 cell cultures was investigated using MTT. 

The results showed no signs of cytotoxicity in Caco-2 cells incubated for 5 h with IC_50_ values greater than 7 mM but concentration-dependent cytotoxicity after 24 h of incubation of nanoparticles with cell culture. The appropriate cell viability data were illustrated in 

Figure 6.

*Permeation studies across Caco-2 cell monolayer*

Transport of insulin in free solution form and also in nanoparticles prepared from non-modified chitosan, trimethyl chitosan, and methylated N-octyl chitosan on Caco-2 cell monolayer was studied. Formation of cell monolayer has been ensured using TEER value and visualization of membrane filter by optical microscopy. The complete cell monolayer has been formed on the trans-well membrane filter after 18 days. The results of permeability studies are illustrated in Figure 7.

As shown in Figure 7, the nanoparticles prepared from methylated N-octyl chitosan cause a significant increase in transports of insulin across the cell monolayer compared to nanoparticles prepared from non-modified chitosan and also to nanoparticle prepared from trimethylchitosan (TMC) (*p < *0.05). 

The increased transport efficiency of the methylated N-octyl derivative is justified by considering the increase in hydrophobicity of the polymer by substituting the octyl derivative which can better interact with intestinal epithelium due to some hydrophobic- hydrophobic interactions.

## Discussion

Using nano-carriers allows one to obtain better and more efficient penetration into the target tissue, and the design of controlled release drug delivery systems. However, despite these advantages, there are limitations in the use of nanoparticles. Their small diameter and high surface to volume ratio leads to the accumulation of particles, and the burst effect of the drug release should be considered (34); but the development of nanoparticles for the delivery of peptides and proteins is considered to be among the most important application of such a delivery system. 

Polymeric nanoparticles can be categorized as natural, semi-natural, or synthetic as to the origin. Comprehensive studies have been done on the application of polymers, such as poly-lactide-co-glycolide (PLGA) (35, 36), chitosan (37- 39) and albumin (40) in peptide delivery. Chitosan can change tight junction flexibility through electrostatic interactions with the intestinal mucosa, and cause an increase in the permeability of the intestinal epithelium (41). In recent years, ionic gelation and PEC have been studied extensively as convenient and efficient techniques for the preparation of nanoparticles containing sensitive molecules, such as peptides and proteins. In these methods, the sonication and use of organic solvents can be avoided, thus damage to the protein structure will be decreased.

It seems that at lower pH, the electrostatic interactions between the polymer and insulin increases, and consequently, compact nanoparticles with low size and high entrapment efficiency will be obtained. At low pH values, the possibility of the protonation of free amines at the polymer backbone increases, resulting in a positively charged polymer, and the strong electrostatic forces between the positively charged polymer and negatively charged insulin leads to the formation of dense and small particles. This can also lead to an increase in the zeta potential of the particles, and consequently, to an increase in drug stability. At higher pH values, due to the lower protonation and thus the decrease in the zeta potential of the particles, the aggregation and formation of nanoparticles with larger sizes increases.

As indicated in Figure 3D, by increasing the pH of the polymer solution, the EE% will be increased until the pH reaches the iso-electric point of the insulin (*i.e.*, 5.6). It has been reported that in the pH values near the iso-electric point of the protein, the ability of the peptides for entrapment into the polymer will be increased (41). By further increase in pH, the positive charge density of the polymer would be reduced and the electrostatic interaction between polymer and insulin is decreased, therefore the EE% is expected to be decreased. 

The size of the nanoparticles was observed to increase by increasing the concentration of the polymer. Since the polymers have a complex structure with a large degree of side branches, it is assumed that the lower concentration of such molecules in the nanoparticles leads to the preparation of smaller particles with lower PdIs.

In this study it is observed that in highest concentration of the polymer, by increasing the insulin concentration, EE% was decreased while in lowest concentration of the polymer, EE% is observed to be increased by increasing the concentration of insulin. Mahjub *et al.* (19) explained that by increasing the concentration of polymer, the rigidity of the polymer would increase and this phenomenon can justify the observed reduction in EE%. 

The stability of the nanoparticles is considered to be one of the more challenging issues in the preparation of these particles. To overcome this problem, the lyophilization technique can be used to obtain solid nanoparticles. To prevent the aggregation, the buildup of sediment particles, and the degradation of protein encapsulated in the polymer, the use of a lyoprotectant is required during the freeze-drying process. The conservation of the particle size is an important factor in a successful lyophilization process. Disaccharides, like sucrose and trehalose, have greater abilities to protect the particle size in comparison with monosaccharides, such as mannitol and glucose. The crystallization of the lyoprotectant may reduce the formation of hydrogen bonds between the lyoprotectant and the polarized active groups of the polymer, and thus, decreases the stability of the particles. According to these studies, sucrose 5% (w/v) has been selected as a lyoprotectant. As observed in this study, although size and PdI of nanoparticles have not changed significantly during lyphilization, but zeta potential, EE%, and LE% of particles were significantly reduced. Mahjub *et al.* (42) previously reported that reduction in EE% and LE% during lyophilization may be attributed to surface drug migration from core of particles and also preparation of some cracks in the surface of particles caused by freeze-drying. 

Based on the data obtained from *in-vitro* release studies, nanoparticles of insulin–chitosan pose a burst effect at the beginning of release, which is lower in quaternized polymers, including the methylated N-octyl chitosan that has been used in this study. This would be due to stronger electrostatic interactions between the polymer and insulin in the methylated derivatives, compared to the non-modified chitosan (20). 

As shown in Figure 7, the cell permeability, and in consequence, oral absorption of the insulin nanoparticles prepared from the methylated N-octyl chitosan is significantly higher than in the nanoparticles prepared by previously studied TMC (*p *< 0.05). It seems that the hydrophobic structure of the octyl derivative improves hydrophobic interactions between nanoparticles and intestinal epithelium, therefore, the paracellular transport of insulin increases. 

Although the permanent positive charge of the quaternized derivatives of chitosan can increase mucoadhesive properties, due to the amphiphilic nature of cell membranes, the presence of a hydrophobic chain that can establish a hydrophobic–hydrophobic interactions between the polymer and membrane can increase the mucoadhesiveness. Based on the data above, both the hydrophobicity and the positive charge play an important role in the development of mucoadhesive characteristics.

In this study, it has been proven that in previously studied aromatic derivatives the non-aromatic, long chain alkylated derivatives of chitosan can also increase the permeability of insulin as one of the large hydrophilic macromolecules across the Caco-2 cell monolayer. 

## Conclusion

In this study, the N, N-dimethyl-N-octyl chitosan was synthesized and used for the preparation of insulin nanoparticles using the PEC method. 

The preparation of the nanoparticles was optimized using the Box-Behnken response surface methodology, while the effects of the variables including the concentration of the polymer solution, pH of the polymer solution, and the concentration of the insulin on the size, zeta potential, PdI, and EE% of the nanoparticles have been investigated.

The morphological studies of the optimized nanoparticles using the SEM revealed uniformly sized spherical nanoparticles with no signs of aggregation. 

An *in-vitro* release study was performed on the nanoparticles under simulated intestinal fluid and the proper release profile of the insulin from the nanoparticles was obtained. The cytotoxicity studies on the Caco-2 cells revealed no significant cytotoxicity for the prepared nanoparticles after 5 h of incubation, but a concentration dependent cytotoxicity was found after 24 h of incubation. The transport studies of the nanoparticles across the Caco-2 cell monolayer revealed significant improvement in the paracellular permeability of the nanoparticles prepared from N,N-Dimethyl-N-Octyl chitosan when compared to trimethyl chitosan. 

The data obtained from this study suggests that the nanoparticles composed of N,N-dimethyl-N-Octyl chitosan are assumed to be good candidates for the oral delivery of hydrophilic macromolecules such as insulin. 
